# Regional Volume Decreases in the Brain of *Pax6* Heterozygous Mutant Rats: MRI Deformation-Based Morphometry

**DOI:** 10.1371/journal.pone.0158153

**Published:** 2016-06-29

**Authors:** Kotaro Hiraoka, Akira Sumiyoshi, Hiroi Nonaka, Takako Kikkawa, Ryuta Kawashima, Noriko Osumi

**Affiliations:** 1 Division of Cyclotron Nuclear Medicine, Cyclotron and Radioisotope Center, Tohoku University, Sendai, Japan; 2 Department of Functional Brain Imaging, Institute of Development, Aging and Cancer, Tohoku University, Sendai, Japan; 3 Department of Developmental Neuroscience, Center for Translational and Advanced Animal Research, Tohoku University Graduate School of Medicine, Sendai, Japan; Northwestern University Feinberg School of Medicine, UNITED STATES

## Abstract

Pax6 is a transcription factor that pleiotropically regulates various developmental processes in the central nervous system. In a previous study, we revealed that *Pax6* heterozygous mutant (*rSey*^*2*^/+) adult rats exhibit abnormalities in social interaction. However, the brain malformations underlying the behavioral abnormality are unknown. To elucidate the brain malformations in *rSey*^*2*^/+ rats, we morphometrically analyzed brains of *rSey*^*2*^/+ and wild type rats using small-animal magnetic resonance imaging (MRI). Sixty 10-week-old rats underwent brain MRI (29 *rSey*^*2*^/+ rats and 31 wild type rats). SPM8 software was used for image preprocessing and statistical image analysis. Normalized maps of the Jacobian determinant, a parameter for the expansion and/or contraction of brain regions, were obtained for each rat. *rSey*^*2*^/+ rats showed significant volume decreases in various brain regions including the neocortex, corpus callosum, olfactory structures, hippocampal formation, diencephalon, and midbrain compared to wild type rats. Among brain regions, the anterior commissure showed significant interaction between genotype and sex, indicating the effect of genotype difference on the anterior commissure volume was more robust in females than in males. The *rSey*^*2*^/+ rats exhibited decreased volume in various gray and white matter regions of the brain, which may contribute to manifestation of abnormal social behaviors.

## Introduction

*Pax6* is a member of the vertebrate paired box (*Pax*) gene family, and has been identified as homologous to the *Drosophila* segmentation gene *paired*. Pax6, a protein product of *Pax6*, is a highly conserved transcription factor among vertebrates and plays important roles in the development of organs including the central nervous system, eyes, nose, pancreas, and pituitary gland. In development of the central nervous system, Pax6 pleiotropically regulates various developmental processes in a highly context-dependent manner through controlling expression of different downstream molecules, including patterning of the neural tube, proliferation and differentiation of neuroepithelial cells, migration of neurons, and formation of neural circuits [1,2].

Human *PAX6* was originally cloned from chromosomal locus 11p13, deletion of which is responsible for WAGR (Wilms tumor, Aniridia, Genital ridge defects, mental Retardation) syndrome [3–5]. Aniridia patients with *PAX6* also show various brain malformations, including absence or hypoplasia of interhemispheric tracts such as the anterior commissure and corpus callosum, decreases in gray matter volume of the cerebellum and occipital pole, hypoplasia of olfactory bulbs, polymicrogyria, and absence of the pineal gland [6–9]. There are case reports showing psychiatric and cognitive disorders such as mental retardation, autism, auditory ineterhemispheric transfer deficits, auditory and verbal working memory deficits, and frontal lobe dysfunction in patients with *PAX6* mutations [10–14].

To explore Pax6 functions, spontaneous *Pax6* mutations were analyzed in mice and rats. Homozygous mutants lack eyes and nose and die soon after birth [15,16], while heterozygous mutants are viable and fertile with slight eye malformations [17]. We previously revealed that *Pax6* heterozygous mutant (*rSey*^*2*^/+) rats exhibited various behavioral abnormalities, such as social interaction defects, impaired sensorimotor gating, and deficits in fear-conditioned memory in adults [18]. The brain malformations in *Pax6* mutants underlying the manifestation of behavioral disorders are unknown, although cortical malformations in heterozygous mutant embryos have been reported at a histological level [19].

Recently, small-animal magnetic resonance imaging (MRI) has been used for structural and functional imaging of experimental animals [20–22]. Statistical image analysis softwares which have been used for human brain studies enable precise voxel-level analysis of brain morphometry in experimental animals. We investigated abnormal morphology in *rSey*^*2*^/+ rats using a deformation-based morphometry (DBM) method. DBM nonlinearly registers a sample brain onto a reference brain to minimize the morphological differences between both brains. The deformation encodes information about the differences as the Jacobian determinant which is finally used to calculate local volume at every voxel [23,24]. We used DBM rather than the voxel-based morphometry (VBM) method because the DBM method detects structural differences independent of sharp tissue borders, whereas the VBM method needs segmentation of the brain into gray and white matters and ventricles and therefore requires high contrast between tissue borders, which is not easy to achieve in imaging the small brain of rodents [24]. In addition to DBM, we used an independent region-of-interest (ROI)-based morphometry method to confirm the results of DBM and to calculate volumes of the ROIs. In independent ROI-based morphometry method, we applied not only parcellation templates in which the whole brain was divided into anatomical regions, but also our previously developed parcellation templates for the neocortex, in which the neocortex was divided into functional regions [25]. The large number of both male and female rats used in this study further enabled the analysis of sex differences in the effect of *Pax6* mutation on brain morphology.

## Materials and Methods

### Animals

Colonies of *rSey*^*2*^/+ rats [26] and wild type (WT) Sprague-Dawley rats were maintained in Tohoku University Graduate School of Medicine. Four pairs of *rSey*^*2*^/+ male rats and WT Sprague-Dawley female rats were mated and litters produced (n = 14 to 18 for each mated pair). The rats were raised in a 12/12 light cycle under temperature- (21–25°C) and humidity- (40–60%) controlled conditions. Food and water were available *ad libitum* throughout the study. Genotypes were judged by mutation-specific polymerase chain reaction (PCR) with genomic DNA extracted from tails [27]. Sixty rats were raised to 10-weeks-old and underwent brain MRI (16 *rSey*^*2*^/+ male, 13 *rSey*^*2*^/+ female, 16 male WT, and 15 female WT rats). All experiments were carried out in accordance with National Institute of Health guidance for the care and use of laboratory animals and were approved by the Committee for Animal Experiments in Tohoku University (No. 2013–005 and 2013–006).

### Image acquisition

Rats were anesthetized with isoflurane (5% for initial induction and 1.5% during MRI scanning) in a gas mixture of 40% O_2_ and 60% N_2_, and all efforts were made to minimize suffering. Each rat was then placed in the prone position on a custom-built MRI bed and its head firmly fixed with a bite bar and gas mask. Respiration rate and core body temperature were continuously monitored using a pressure sensor attached to the abdominal area and an MRI-compatible temperature probe inserted into the rectum (Model 1025, SA Instruments, Stony Brook, NY, USA). Core body temperature was maintained at 37.0 ± 1.0°C throughout the scan using a water-circulating pad. MRI data were acquired using a 7.0 T Bruker PharmaScan system (Bruker Biospin, Ettlingen, Germany) with a 38 mm diameter birdcage coil (Bruker Biospin, Ettlingen, Germany) with eight rods and two end rings designed for imaging the rat brain. Prior to data acquisition, global magnetic field was shimmed inside the core and at region of interest (ROI) using a point-resolved spectroscopic protocol. Line width (full width at half maximum) at the end of the shimming ranged from 15 to 20 Hz in the ROI (~300 μl). We used T2-weighted images for the analysis because T1 tissue contrast between gray and white matters is less clear in a high magnetic field strength in rodents than humans [28]. T2-weighted images were obtained using the respiration-gated two-dimensional rapid acquisition of relaxation enhancement (2D-RARE) sequence with the following parameters: TR = 4800 ms, TEeff = 30 ms, RARE factor = 4, FOV = 32 × 32 mm^2^, matrix size = 256 × 256, voxel size = 125 × 125 μm^2^, number of slices = 56, slice thickness = 0.5 mm, slice gap = 0 mm, and number of averages = 8. Total MRI scanning time for each rat was approximately 60 min, dependent on respiration rate. After MRI imaging, rats were sacrificed with overdose pentobarbital. We chose 0.5-mm thickness because an isotropic T2 imaging with thickness of 0.125 mm is not a possible scanning condition for in vivo imaging, which takes more than six hours per an animal. Indeed, previous studies succeeded in morphologically analyzing the brain of rodents with slice thickness of 0.5 to 1.0 mm [29–31]. Signal-to-noise ratio of the images was 26 ± 2 (mean ± standard deviation), which was measured as the mean image intensity in a single slice of the brain divided by the standard deviation of the intensity in the background outside the brain.

### Deformation-based morphometry (DBM)

The statistical parametric mapping software SPM8 (Wellcome Trust Centre for Neuroimaging, London, UK) was used for image preprocessing and statistical image analysis. First, each T2-weighted image was resized by a factor of 10 to account for the whole brain volume difference between human and rodent [32], and the rigid body was aligned to the stereotaxic space by registering each image to an in vivo rat T2 template image [25] and re-sampled into 1.25 mm isotropic voxels (for the resized images). Second, each T2-weighted image was segmented into probability maps of gray matter (GM), white matter (WM), and cerebrospinal fluid (CSF) using the unified segmentation approach which enables image registration, tissue classification, and bias correction to be combined within the unified generative model [33]. For the unified segmentation steps, the default settings in the SPM8 toolbox were used, except human tissue priors were replaced by rat tissue priors [25]. Third, the obtained GM, WM, and CSF images were used to create a customized, more population-specific template using diffeomorphic anatomical registration using the exponentiated lie algebra (DARTEL) template-creation tool [34]. Lastly, the Jacobian determinants (a parameter for the expansion and/or contraction) at each voxel within the brain were calculated using the deformation fields from the DARTEL algorithm and finally smoothed using an isotropic Gaussian kernel of 10 mm for the resized images. Although image preprocessing was performed in the resized scales, the results of image analysis were displayed in the original scales.

We tested for group-wise differences in the Jacobian determinant values in each voxel using a 2-by-2 analysis of variance (ANOVA) in the SPM8 toolbox, with genotype and sex as factors. Statistically significant clusters were identified using a threshold of 500 voxels and *p* < 0.05, after which a family-wise error (FWE) correction for multiple comparisons was applied across all brain voxels.

**Fig 1:**
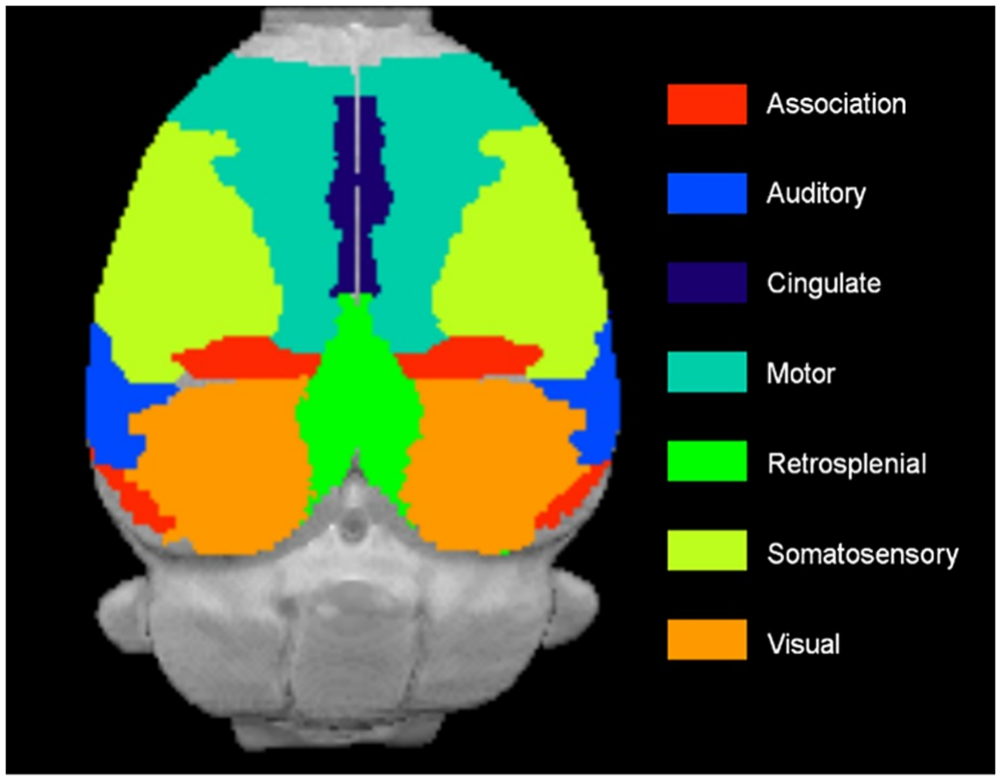
Cortical regions superimposed on the T2-weighted MRI template. Insular region is ventral and not shown in the figure.

To clarify which regions included the significant voxels, the number of significant voxels in brain and neocortical regions was counted using parcellation templates for the whole brain, in which the whole brain is divided into anatomical regions [35], and for the neocortex, in which the neocortex is divided into functional regions [25]. These parcellation templates were nonlinearly registered to the population-specific template space. In the parcellation templates for the whole brain, the brain was segmented into 26 regions (Table 1). In the original templates of the neocortex, the neocortex was segmented into 48 regions per hemisphere. In our analysis, the neocortex was segmented into nine regions based on the original template (Fig 1). The regions in the original templates included in our analysis are shown in S1 Table.

**Table 1:** Number of voxels in each region which showed significant volume decrease in *rSey*^*2*^/+ rats compared to WT rats in voxel-by-voxel analysis (FWE, p < 0.05; threshold of 500 voxels).

	All *rSey*^*2*^*/+* (n = 29) vs. all WT (n = 31)		Female *rSey*^*2*^*/+* (n = 13) vs. female WT (n = 15)		Male *rSey*^*2*^*/+* vs. (n = 16) male WT (n = 16)	
	Number of significant voxels	%	Number of significant voxels	%	Number of significant voxels	%
Accumbens nucleus	1	0.0	0	0.0	0	0.0
Amygdala	3856	2.7	586	1.3	0	0.0
Anterior commissure	170	0.1	132	0.3	0	0.0
Bed nucleus of the stria terminalis	0	0.0	0	0.0	0	0.0
Cerebellum	0	0.0	0	0.0	0	0.0
Cingulum	1848	1.3	1630	3.5	587	4.2
Corpus callosum	14115	10.0	9169	19.7	3385	24.0
Diagonal domain	2	0.0	0	0.0	0	0.0
Diencephalon	14141	10.0	728	1.6	0	0.0
Fimbria	71	0.1	0	0.0	0	0.0
Hindbrain	0	0.0	0	0.0	0	0.0
Hippocampal formation	6564	4.7	1837	4.0	757	5.4
Hypothalamus	349	0.2	83	0.2	0	0.0
Internal capsule	971	0.7	33	0.1	0	0.0
Neocortex	84608	60.0	28531	61.4	9258	65.6
Midbrain	6558	4.6	45	0.1	135	1.0
Olfactory structures	6928	4.9	3563	7.7	0	0.0
Optic pathways	426	0.3	145	0.3	0	0.0
Pallidum	0	0.0	0	0.0	0	0.0
Pineal gland	0	0.0	0	0.0	0	0.0
Pituitary	0	0.0	0	0.0	0	0.0
Preoptic area	0	0.0	0	0.0	0	0.0
Septum	0	0.0	0	0.0	0	0.0
Striatum	0	0.0	0	0.0	0	0.0
Substantia nigra	12	0.0	0	0.0	0	0.0
Ventricles	457	0.3	19	0.0	0	0.0
Sum	141077	100	46501	100	14122	100

FWE, family wise error; *rSey*^*2*^*/+*, Pax6 heterozygous mutant; WT, wild-type

### Independent ROI-based morphometry

In addition to the abovementioned DBM analysis, we performed an independent ROI-based morphometry analysis based on rat MRI templates of the whole brain [35] and neocortex [25]. First, each T2-weighted image was resized by a factor of 10, skull-stripped, and bias-corrected. Second, the rigid body was aligned to the template image and re-sampled into 1.25 mm isotropic voxels (for the resized images). Third, the image was normalized to the template image using the “Normalize” function in the SPM8 toolbox, providing the transformation matrix of the template space. ROI images of the 26 brain structures and nine neocortical regions in the template space were then inversely normalized to the individual space based on the transformation matrix obtained above. Finally, volumes in each anatomical structure were obtained in the individual space for each rat. In addition to volumes of the 26 brain regions and nine neocortical regions, volumes of the total GM, total WM, and CSF were calculated using GM, WM, and CSF images in native space, obtained in the process of DBM. Group-wise differences in volumes of each region were tested using a 2-by-2 analysis of variance (ANOVA) in IBM SPSS statistics 22 statistical analysis software (IBM Corp., New York, USA) with genotype and sex as factors. The statistical significance was set at *p* < 0.05. The significance level for multiple comparisons was not corrected because of the explorative nature of this study.

## Results

### Analyses by DBM

**Fig 2:**
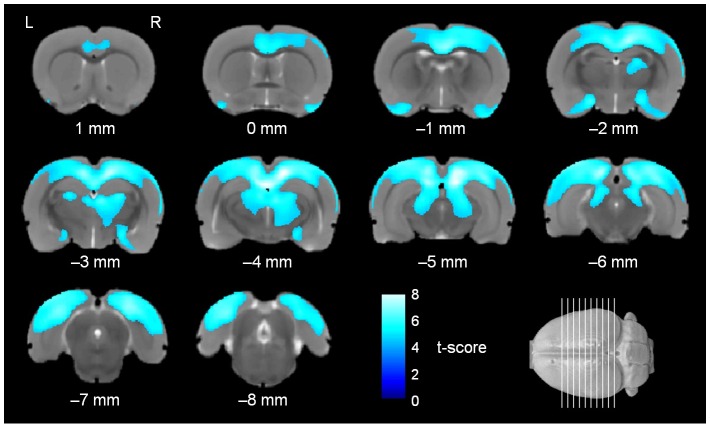
Regional volume decrease in the brain of *rSey*^*2*^/+ rats compared to WT rats. Colored voxels represent clusters of significant regional volume decrease in the brain of *rSey*^*2*^/+ rats (n = 29) compared to WT rats (n = 31) in ANOVA (FWE, *p* < 0.05; threshold of 500 voxels) superimposed on the T2-weighted MRI template. rSey^2^/+, Pax6 heterozygous mutant; WT, wild-type; ANOVA; analysis of variance; FWE, family-wise error.

The voxel-wise 2-by-2 ANOVA with genotype and sex as factors of the Jacobian determinant values revealed voxels with significant volume decreases (FWE-corrected, *p* < 0.05) widely distributed across the brain. The significant voxels are superimposed on the population-specific T2-weighted template in Fig 2. The number of voxels with significant volume decreases in each region was counted using the parcellation template and is shown in Table 1. The *rSey*^*2*^/+ rats showed significant volume decreases in various brain regions, including the amygdala, anterior commissure, cingulum, corpus callosum, diencephalon, fimbria, hippocampal formation, hypothalamus, internal capsule, neocortex, midbrain, olfactory structures, and optic pathways, compared to WT rats. In analysis of the neocortex, the number of voxels with significant volume decreases in *rSey*^*2*^/+ rats compared to WT rats in each neocortical region was counted using the parcellation template of the neocortex and is shown in Table 2. Significant voxels were observed in all cortical regions. Among the neocortical regions, the visual area contained most of the voxels with significant volume decreases (32.1% of significant voxels). There were no voxels in the brain that showed significant volume increases in *rSey*^*2*^/+ rats compared to the WT rats. No interaction between genotype and sex was detected in the DBM. These results suggest that *rSey*^*2*^/+ rats showed significant volume decreases in the various gray and white matter regions.

**Table 2:** Number of voxels in each neocortical region which showed significant volume decrease in *rSey*^*2*^/+ rats compared to WT rats in voxel-by-voxel analysis (FWE, p < 0.05; threshold of 500 voxels).

	All *rSey*^*2*^*/+* (n = 29) vs. all WT (n = 31)		Female *rSey*^*2*^*/+* (n = 13) vs. female WT (n = 15)		Male *rSey*^*2*^*/+* (n = 16) vs. male WT (n = 16)	
	Number of significant voxels	%	Number of significant voxels	%	Number of significant voxels	%
Insular	28	0.0	0	0.0	0	0.0
Auditory	4776	5.7	727	2.6	5	0.1
Cingulate	6038	7.2	2655	9.4	0	0.0
Motor	6830	8.2	3144	11.1	419	4.4
Retrosplenial	10011	12.0	3298	11.6	2505	26.1
Somatosensory	15659	18.8	1753	6.2	0	0.0
Visual	26745	32.1	11627	41.0	6041	62.9
Association	8652	10.4	4169	14.7	346	3.6
Other regions	4644	5.6	971	3.4	290	3.0
Sum	83383	100	28344	100	9606	100

family-wise error; *rSey*^*2*^*/+*, Pax6 heterozygous mutant; WT, wild-type

**Fig 3:**
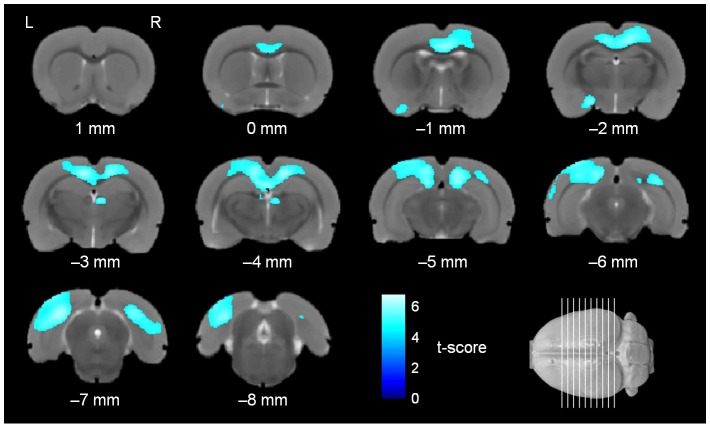
Regional volume decrease in the brain of female *rSey*^*2*^/+ rats compared to female WT rats. Colored voxels represent clusters of significant regional volume decrease in the brain of female *rSey*^*2*^/+ rats (n = 13) compared to female WT rats (n = 15) in the post hoc analysis after ANOVA (FWE, *p* < 0.05, threshold of 500 voxels), superimposed on the T2-weighted MRI template. rSey^2^/+, Pax6 heterozygous mutant; WT, wild-type; ANOVA; analysis of variance; FWE, family-wise error.

**Fig 4:**
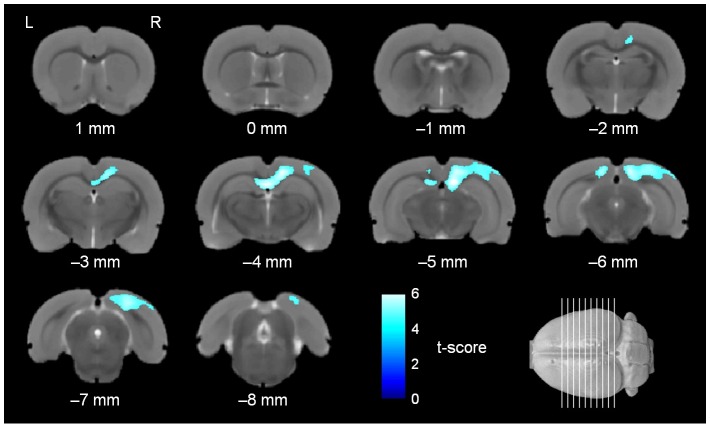
Regional volume decreases in the brain of male *rSey*^*2*^/+ rats compared to male WT rats. Colored voxels represent clusters of significant regional volume decreases in the brain of male *rSey*^*2*^/+ rats (n = 16) compared to male WT rats (n = 16) in the post hoc analysis after ANOVA (FWE, *p* < 0.05, threshold of 500 voxels), superimposed on the T2-weighted MRI template. rSey^2^/+, Pax6 heterozygous mutant; WT, wild-type; ANOVA; analysis of variance; FWE, family-wise error.

In the post hoc analysis, comparisons between female *rSey*^*2*^/+ (n = 13) and female WT (n = 15) rats and between male *rSey*^*2*^/+ (n = 16) and male WT (n = 16) rats were performed. The significant voxels were superimposed on the population-specific T2-weighted template in Figs 3 and 4. Female and male *rSey*^*2*^/+ rats showed similar pattern of volume decrease in the brain. However, as shown in Table 1, the number of voxels showing significant volume decreases was much larger in female *rSey*^*2*^/+ rats compared to female WT rats than the number in male *rSey*^*2*^/+ rats compared to male WT rats. In the neocortical analysis, the visual region showed the largest number of significant voxels in both female and male *rSey*^*2*^/+ rats (Table 2). No voxels showed significant volume increases in *rSey*^*2*^/+ rats compared to WT rats in post hoc analysis. Although interaction between genotype and sex was undetected in the DBM, post hoc analysis indicated that the regions showing significant volume decreases in female *rSey*^*2*^/+ rats compared to female WT rats were wider than the regions showing significant volume decreases in male *rSey*^*2*^/+ rats compared to male WT rats, suggesting a possibility that the effect of *Pax6* mutation on the brain volume is more prominent in females than in males.

### Analyses by independent ROI-based morphometry

The *rSey*^*2*^/+ rats (n = 29) showed significant volume decrease in the total gray and white matters respectively compared to WT rats (n = 31) (Table 3). The *rSey*^*2*^/+ rats showed a 3.0% volume decrease in total gray matter and a 3.4% volume decrease in total white matter compared to WT rats. The *rSey*^*2*^/+ rats also showed significant volume decreases in various brain regions, i.e., the amygdala, anterior commissure, cingulum, corpus callosum, diencephalon, fimbria, hippocampal formation, internal capsule, neocortex, midbrain, olfactory structures, optic pathways, pallidum and pineal gland compared to WT rats (Table 3). Most of the results were consistent with those in DBM. However, the pallidum and pineal gland showed significant volume decrease in independent ROI-based morphometry, although there were no voxels which showed significant volume decrease in these regions in DBM (Table 1). The diagonal domain showed significant volume increase in independent ROI-based morphometry, although there were no voxels which showed significant volume increase in the region in DBM. On the other hand, a small number of voxels in the accumbens nucleus, hypothalamus, and substantia nigra showed significant volume decrease in DBM (Table 1), although they did not show significant volume decrease in independent ROI-based morphometry. In the neocortex, all cortical areas showed significant volume decreases in *rSey*^*2*^/+ rats compared to WT rats (Table 4). Above all, the visual area (i.e., the posterior region of the neocortex) showed the largest volume decrease (5.7%). Among the ROIs, only the anterior commissure showed a statistically significant interaction between genotype and sex [F(1, 56) = 4.881, *p* < 0.05], indicating that the effect of genotype differences on the anterior commissure volume was more robust in females than in males (Table 3).

**Table 3:** Volumes of brain regions in the ROI analysis.

	*rSey*^*2*^*/+* rats (all)		WT rats (all)		Difference (%)	Main effect of genotype		Interaction between genotype and sex	
	mean (μl)	SD (μl)	mean (μl)	SD (μl)		*F*		*F*	
Gray matter	1103.6	73.6	1137.8	68.1	-3.0	8.08	**	0.27	
White matter	566.9	33.2	586.7	23.8	-3.4	9.24	**	0.53	
CSF	144.7	19.8	145.8	17.4	-0.7	0.30		0.72	
Sum of gray and white matter and CSF	1815.3	115.7	1870.2	98.3	-2.9	8.70	**	0.54	
Accumbens nucleus	12.1	0.9	12.2	0.8	-1.0	0.72		0.55	
Amygdala	39.2	2.6	40.9	2.5	-4.0	15.50	***	0.04	
Anterior commissure	3.0	0.2	3.3	0.2	-8.6	41.46	****	4.88	*
Bed nucleus of the stria terminalis	2.4	0.2	2.4	0.2	0.5	0.00		0.55	
Cerebellum	281.5	23.1	282.7	18.5	-0.4	0.25		0.19	
Cingulum	4.5	0.4	4.8	0.3	-7.1	28.95	****	0.68	
Corpus callosum	67.6	5.1	72.4	3.6	-6.7	25.65	****	1.22	
Diagonal domain	4.6	0.2	4.5	0.2	3.0	6.27	*	0.08	
Diencephalon	97.7	5.7	102.6	4.8	-4.8	19.81	****	0.43	
Fimbria	14.4	1.0	14.8	0.8	-3.2	6.49	*	1.11	
Hindbrain	195.0	8.6	193.8	7.1	0.6	0.27		0.01	
Hippocampal formation	98.8	8.6	101.9	7.4	-3.0	6.15	*	0.21	
Hypothalamus	34.0	2.1	34.4	1.7	-1.3	2.48		0.91	
Internal capsule	27.6	1.6	28.7	1.3	-3.8	11.82	**	0.44	
Neocortex	579.7	35.1	605.5	32.1	-4.3	21.13	****	0.44	
Midbrain	86.2	5.9	89.8	5.4	-4.0	8.85	**	0.04	
Olfactory structures	114.4	5.3	117.9	4.8	-3.0	14.80	***	1.81	
Optic pathways	3.7	0.2	3.9	0.2	-3.5	11.91	**	0.99	
Pallidum	14.9	1.0	15.5	0.8	-3.7	9.19	**	0.95	
Pineal gland	1.8	0.2	1.9	0.2	-7.5	11.54	**	0.18	
Pituitary	10.2	0.4	10.2	0.4	0.2	0.11		0.62	
Preoptic area	8.0	0.6	7.9	0.5	1.0	0.17		0.85	
Septum	12.6	0.9	12.6	0.7	0.0	0.05		1.05	
Striatum	80.9	7.2	83.5	7.1	-3.1	3.28		1.51	
Substantia nigra	6.0	0.4	6.1	0.3	-2.5	3.37		0.01	
Ventricles	6.9	1.0	6.8	0.7	1.1	0.02		1.21	

Two-way ANOVA:

*p < 0.05,

**p < 0.01,

***p < 0.001,

****p < 0.0001

ROI, region-of-interest; *rSey*^*2*^*/+*, Pax6 heterozygous mutant; WT, wild-type; SD, standard deviation; CSF, cerebrospinal fluid

**Table 4:** Volumes of the neocortical regions in the ROI analysis.

	*rSey*^*2*^*/+* rats (all)		WT rats (all)		Difference (%)	Main effect of genotype		Interaction between genotype and sex
	mean (μl)	SD (μl)	mean (μl)	SD (μl)		*F*		*F*
Insular	41.8	2.6	43.3	2.6	-3.6	10.78	**	0.64
Auditory	35.6	2.5	37.6	2.3	-5.3	18.13	****	0.42
Cingulate	34.3	2.1	35.9	1.8	-4.3	19.31	****	0.44
Motor	71.9	4.9	74.8	4.2	-3.9	12.76	***	0.64
Retrosplenial	32.3	2.0	34.2	2.1	-5.4	34.90	****	0.32
Somatosensory	129.0	7.6	135.3	7.1	-4.6	19.97	****	0.53
Visual	56.2	3.0	59.6	3.0	-5.7	52.06	****	0.01
Association	18.6	1.1	19.7	1.0	-5.5	41.53	****	0.44
Other regions	89.0	5.3	91.7	5.0	-2.9	11.11	**	0.06

Two-way ANOVA:

*p < 0.05,

**p < 0.01,

***p < 0.001,

****p < 0.0001

ROI, region-of-interest; *rSey*^*2*^*/+*, Pax6 heterozygous mutant; WT, wild-type; SD, standard deviation; CSF, cerebrospinal fluid

Since we scanned a relatively large amount of samples, we next analyzed the brain imaging data by dividing it into male and female subgroups. In post hoc analysis, comparisons between female *rSey*^*2*^/+ (n = 13) and female WT (n = 15) rats and between male *rSey*^*2*^/+ (n = 16) and male WT (n = 16) rats were performed. The results are shown in S2 and S3 Tables. Total gray and white matter volumes both showed significant decreases in female *rSey*^*2*^/+ rats compared to female WT rats, whereas they did not show significant volume decreases in male *rSey*^*2*^/+ rats compared to male WT rats (S2 Table). The number of ROIs showing a significant volume decrease in female *rSey*^*2*^/+ rats was larger than that in male *rSey*^*2*^/+ rats. In the neocortical analysis, female *rSey*^*2*^/+ rats showed a significant volume decrease compared to female WT rats in all neocortical regions, whereas male *rSey*^*2*^/+ rats showed a significant volume decrease compared to male *rSey*^*2*^/+ rats in all neocortical regions but the insular region (S3 Table). Again, post hoc analysis suggested that the effect of *Pax6* mutation on brain volume is more prominent in females than in males.

## Discussion

Although MRI study on cortex-specific *Pax6* knockout mice reported reduced cortical thickness and disorganization of callosal fibers [36], this is the first study that analyzed the brain morphology of rodents with spontaneous *Pax6* mutation. Our method enabled precise morphometry of each brain region and neocortical subregion. We used 7 tesla magnetic resonance (MR) scanner because 4.7 to 11.7 tesla MR scanners are standard modalities used for brain imaging studies of rodents [35,37–39] although 7 Tesla or higher MR imaging has been applied to human subjects. Similarly, a birdcage coil is usually used in brain imaging studies of rodents [39,40]. Human subjects with PAX6 mutations showed various psychiatric and cognitive disorders. In accordance with the human subjects, Pax6 heterozygous mutant rats manifest abnormal social behaviors [18], and we think that it is worth analyzing the brain abnormal morphology which may underlie the behaviors. Our MRI analyses revealed severe decreases in the volumes of various gray and white matter regions of the *rSey*^*2*^/+ rat brain compared to the WT rat brain. The DBM and independent ROI-based morphometry consistently showed decreased volume in the brain regions including the neocortex, cingulum, corpus callosum, olfactory structures, amygdala, hippocampal formation, diencephalon, and midbrain in *rSey*^*2*^/+ rats compared to WT rats. Both morphometry showed consistent results in most of the brain regions except the accumbens nucleus, diagonal domain, hypothalamus, pallidum, pineal gland, and substantia nigra. We could not compare the accuracy of DBM and independent ROI-based morphometry because DBM does not quantitatively analyze volumes. Although it is not quantitative, DBM can reveal statistical volume change in each voxel. ROI-based morphometry cannot analyze volumes voxel by voxel but it can quantify volumes of ROIs. We used the both methods because those complement each other and reinforce reliability of each other.

### Pax6-expressing regions at embryonic and adult stage and regions with volume decrease in the adult mutant rats

**Fig 5:**
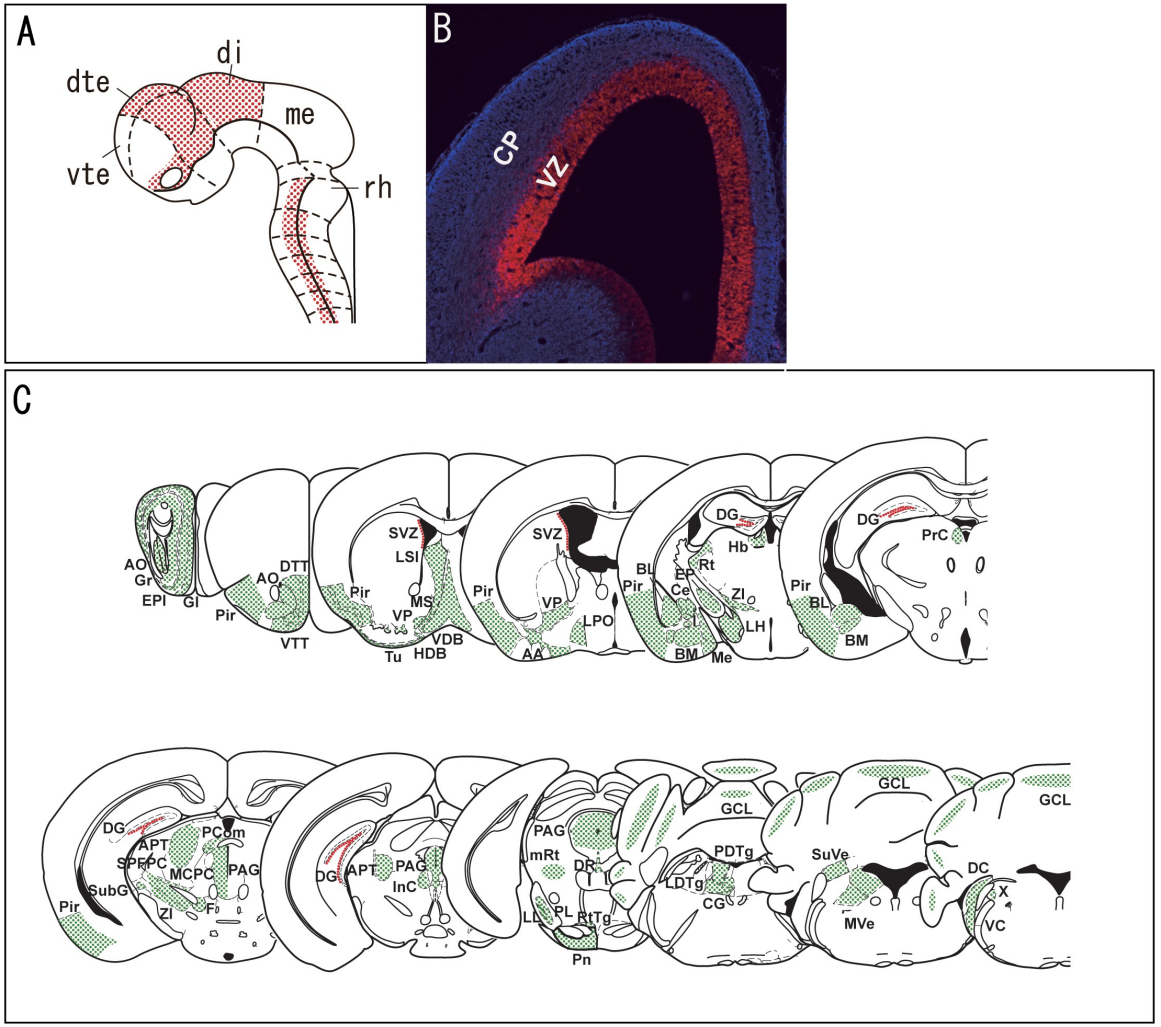
**(A) Expression pattern of *Pax6* in rat embryo at E10.5.** The figure was modified from Kikkawa et al. [50]. dte, dorsal telencephalon; di, diencephalon; me, mesencephalon; rh, rhombencephalon; vte, ventral telencephalon. **(B) Pax6 expression in an E14.5 mouse cortex.** Pax6 protein (red) is restricted to the ventricular zone (VZ), where neural progenitor cells reside; in contrast, *Pax6* is not expressed in the Tuj1-positive (blue) cortical plate (CP). The figure was modified from Osumi and Kikkawa [51]. **(C) Expression pattern of *Pax6* in the adult mouse based on previously published data (Duan et al., 2013** [41]; **Haba et al., 2009** [52]; **Kohwi et al., 2005** [53]; **Maekawa et al., 2005** [54]). The regions with *Pax6* expression in the studies of Duan et al. and Haba et al. are shown in green, and the regions in which *Pax6* was expressed in neural stem cells/progenitor cells in the other studies are shown in red. Nomenclature and illustration of various brain regions are based on *The Mouse Brain in Stereotaxic Coordinates* (2^nd^ Edition) [55]. AA, anterior amygdaloid area; AO, anterior olfactory nucleus; APT, anterior pretectal nucleus; BM/BL, basomedial/basolateral amygdaloid nucleus; Ce, central amygdaloid nucleus; CG, central gray; DG, hippocampal dentate gyrus; DR, dorsal raphe nucleus; EP, entopeduncular nucleus; EPl, external plexiform layer of the olfactory bulb; F, nucleus of the fields of Forel; GCL, granular cell layer of the cerebellum; Gl, glomerular layer of the olfactory bulb; Gr, granule cell layer of the olfactory bulb; Hb, habenular nucleus; I, intercalated nuclei of the amygdala; InC, interstitial nucleus of Cajal; LH, lateral hypothalamic area; LL, nucleus of the lateral lemniscus; LPO, lateral preoptic area; LSI, lateral septal nucleus, intermediate part; MCPC, magnocellular nucleus of the posterior commissure; Me, Medial amygdaloid nucleus; mRt, mesencephalic reticular formation; MS, medial septal nucleus; PAG, periaqueductal gray; PCom, nucleus of the posterior commissure; PDTg/LDTg, posterodorsal/laterodorsal tegmental nucleus; Pir, Piriform cortex; PL, paralemniscal nucleus; Pn, pontine nuclei; PrC, precommissural nucleus; Rt, reticular thalamic nucleus; RtTg, reticulotegmental nucleus of the pons; SubG, subgeniculate nucleus; SuVe/MVe, superior/medial vestibular nucleus; SPFPC, subparafascicular thalamic nucleus, parvicellular part; SVZ, subventricular zone; Tu, olfactory tubercle; VC/DC, ventral/dorsal cochlear nucleus; VDB/HDB, nucleus of the vertical/horizontal limb of the diagonal band; VP, ventral pallidum; VTT/DTT, ventral/dorsal tenia tecta; X, nucleus X; ZI, zona incerta.

Most of the brain regions which showed volume decrease in the Pax6 heterozygous mutant rats, i.e., the amygdala, diencephalon, hippocampal formation, neocortex, midbrain, olfactory structures, pallidum, and pineal gland express Pax6 at embryonic and/or adult stages (Fig 5) [41–43]. The anterior commissure, cingulum, corpus callosum, fimbria, internal capsule, and optic pathways showed volume decrease, although Pax6 does not seem to be expressed in these regions. However, we observe Pax6 expression in oligodendrocyte precursor cells in our unpublished preliminary study. Therefore, the volume decrease of these white matter structures may be due to abnormal proliferation or differentiation of oligodendrocyte lineages, to non-autonomous effect of other brain regions such as the neocortex and diencephalon that project axons to these white matter structures, and/or to abnormal pathfinding of the axons in mutant rats. The volume decrease of the anterior commissure and corpus callosum in *rSey*^*2*^/+ rats seems to be a milder phenotype considering the almost complete lack of interhemispheric connectivity observed in cortex-specific *Pax6* knockout mice [36]. Intriguingly, human subjects with *PAX6* mutations also show hypoplasia or aplasia of these structures [6–9,44,45] and manifest auditory interhemispheric transfer deficits as a result of interhemispheric-fiber dysgenesis [45]. On the other hand, the cerebellum, hindbrain, and septum did not show volume decrease despite Pax6 expression in these regions. Normal cerebellar volume is consistent with our previous study that showed normal locomotor activity in *Pax6* heterozygous mutant rats [18], although it has been suggested that Pax6 plays a critical role in cerebellar granule cell development at embryonic and adult stages [46–48]. We could not clarify the reason why the volume decrease was not observed in the hindbrain instead of a critical role of Pax6 in development of it [26,49]. The relation between Pax6 and development of the septum has not been elucidated yet.

### The roles of Pax6 and volume decrease in the mutant rats

It is no wonder that *Pax6* mutation affects various regions of the brain because *Pax6* is expressed throughout the central nervous system during embryonic development (Fig 5A). P*ax6* is expressed in neural stem/progenitor cells first (Fig 5B, [2]). Since *Pax6* regulates neurogenesis of neural stem/progenitor cells [2], insufficient regulation of proliferation and differentiation during brain development of *rSey*^*2*^/+ rats may inevitably cause decreased numbers of neuronal cells, resulting in decreased volume, especially of the gray matter. *Pax6* also non-cell-autonomously regulates axonal extension in the embryonic stage; *Pax6* expression guides thalamocortical tract formation [56] and projection from the substantia nigra to the ventral tegmental area [57]. Misregulation of axonal extension during brain development of *rSey*^*2*^/+ rats may therefore cause shortening of axons, resulting in volume decreases, especially in the white matter.

In the adult, *Pax6* expression continues in neural stem/progenitor cells in neurogenic regions such as the subventricular zone of the lateral ventricle and the subgranular zone of the hippocampus. The former is required for making specific subpopulations of granule and periglomerular neurons in the olfactory bulb [53] and the latter for production of granule cells in the hippocampus [54]. Therefore, decreases in the volume of the olfactory structures and hippocampal formation of *rSey*^*2*^/+ rats may be attributed by the impaired neurogenesis in the adult. Moreover, in adults, *Pax6* expression is observed in various other regions (Fig 5C), and it is possible that adult expression of *Pax6* in the regions may affect morphology of the brain in *rSey*^*2*^/+ rats.

In addition to the MRI analysis, we briefly examined the histology of the brain of *rSey*^*2*^/+ rats using Kluver-Barrera staining and a light microscope to elucidate histological mechanisms underlying volume decrease in various regions of the brain (data not shown). However, we did not find remarkable histological changes, although heterozygous mice with a mutation at the *Pax6* locus (*Sey*^*Neu*^*/+*) show apparent histological abnormalities such as hypoplasia of the telencephalic frontal area in the embryo [19]. There remains a possibility that histological abnormalities can be found in *rSey*^*2*^/+ rats if specific regions are investigated in specific ways, e.g., immunohistological analysis of specific types of interneurons in specific cortical layers.

### Area difference of volume decrease in the neocortex

In the analysis of the neocortex, the degree of volume decrease in caudal regions (the auditory, retrosplenial, visual, and association regions) was more prominent than that in rostral regions (the insular, cingulate, motor, and somatosensory regions). The visual region showed a 5.7% volume decrease, the largest degree of volume decrease among the neocortical regions. This was rather unexpected because *Pax6* homozygous mutant mice (*Sey/Sey*) show abnormal neocortical arealization; rostro-lateral areas such as the motor cortex show shrinkage, while caudo-medial areas such as the visual cortex show expansion [58]. There may thus be other reasons for the volume reduction in the visual cortex.

Cortical circuits can be modified by manipulations such as perceptual learning and visual deprivation. The mammalian visual cortex has experience-dependent plasticity not only in the developmental stage but also in the adult stage [59]. Moreover, the volume of the visual cortex is decreased in patients with visual dysfunctions due to amblyopia or glaucoma [60,61]. Potential visual impairment in spontaneous *Pax6* mutant rodents due to eye abnormalities [18,26] may similarly result in volume reduction in the visual cortex. In interpreting the results of neocortical analysis, we must remember that neocortical parcellation templates can be misaligned to the neocortex of *rSey*^*2*^/+ rats because *Pax6* mutation does affect neocortical arealization [62,63].

### Brain volume decrease and abnormal social behaviors in *rSey*^*2*^/+ rats

Research for the neural correlates of social behaviors has pointed to several systems that process sensory information relevant to social interactions [64,65]. In rodents, these systems can be roughly divided into two modules. One is a subcortical circuit that carries olfactory social information from the accessory olfactory bulb to the amygdala, which modulates hypothalamic activity to regulate behavioral responses. The other consists of cortical regions like the prefrontal cortex that provide top-down control over the activity of subcortical circuits. Because *rSey*^*2*^/+ rats showed volume decreases in these regions relevant to social behaviors, malformation of all or either of these regions can contribute to abnormal social behaviors.

We posit that *rSey*^*2*^/+ rats could be an animal model for autism spectrum disorders because we have already reported abnormal behaviors, including social abnormalities, in *rSey*^*2*^/+ rats [18], and defect in social interactions is one of the core features of such disorders [66]. Recently, a new gene encoding the adhesive junction-associated delta-catenin protein (*CTNND2*) has been identified as responsible for manifestation of the symptoms from genetic analysis of female subjects with severe autism [67]. In the study, the authors discuss the possible involvement of *PAX6* in regulation of *CTNND2* because expression of delta-catenin is downregulated in the *Pax6* homozygous mutant mouse cortex and retina [68]. Our preliminary data also showed downregulation of delta-catenin in the developing cortex of *rSey*^*2*^/ *rSey*^*2*^ (unpublished data). Our study may therefore contribute to elucidating the mechanisms underlying manifestation of autism spectrum disorders.

### Sex difference in the effect of *Pax6* mutation on brain volume

Independent ROI-based morphometry detected interaction between genotype and sex in the anterior commissure, indicating that the effect of genotype differences on the anterior commissure volume was more robust in females than in males. In addition, more prominent volume decreases were observed in female *rSey*^*2*^/+ rats than in male *rSey*^*2*^/+ rats in the *post hoc* analyses comparing female *rSey*^*2*^/+ vs. WT rats and male *rSey*^*2*^/+ vs. WT rats. In our previous behavior analyses, we have found that the frequency of an isolated pup’s mother calls was significantly decreased in female *rSey*^*2*^/+ pups compared to female WT pups, whereas male *rSey*^*2*^/+ pups did not show a significant difference from male WT [18]. More severe volume decreases in the brain of female *rSey*^*2*^/+ rats may relate to sex-biased abnormalities in behavior levels, although we have not analyzed behavioral differences between male and female rats as adults. Future analyses of behavior in *Pax6* female mutant rats/mice would be interesting in light of the more severe symptoms often observed in female subjects with autism.

## Conclusions

The *rSey*^*2*^/+ rats showed decreased volume in various gray and white matter regions of the brain, which may contribute to that manifestation of abnormal social behaviors. The mechanisms underlying the volume decrease and its contribution to the abnormal behaviors in *rSey*^*2*^/+ rats are to be clarified in future studies. Further study is also needed to clarify the possibility of a sex difference in the effect of Pax6 mutation on the brain. These studies may shed light on pathogenic mechanism in developmental disorders, especially autism spectrum disorders.

## Supporting Information

S1 Table Subregions included in the neocortical regions. (DOCX)

S2 Table Volumes of brain regions in the ROI analysis. (DOCX)

S3 Table Volumes of neocortical regions in the ROI analysis. (DOCX)
