# Supplementary material for: Regional Volume Decreases in the Brain of Pax6 Heterozygous Mutant Rats: MRI Deformation-Based Morphometry
Source: PLoS One. 2016 Jun 29;11(6):e0158153. doi: 10.1371/journal.pone.0158153 (PMC4927189; doi:10.1371/journal.pone.0158153)
Supplement: S1 Table — (DOCX) [file pone.0158153.s001.docx]

| Insular | agranular insular cortex, dorsal part | Visual | primary visual cortex |
| --- | --- | --- | --- |
|  | agranular insular cortex, posterior part |  | primary visual cortex, binocular area |
|  | agranular insular cortex, ventral part |  | primary visual cortex, monocular area |
|  | dysgranular insular cortex |  | secondary visual cortex, lateral area |
|  | granular insular cortex |  | secondary visual cortex, mediolateral area |
|  | dysgranular insular cortex |  | secondary visual cortex, mediomedial area |
| Auditory | primary auditory cortex | Association | lateral parietal association cortex |
|  | secondary auditory cortex, dorsal area |  | medial parietal association cortex |
|  | secondary auditory cortex, ventral area |  | temporal association cortex |
| Cingulate | cingulate cortex, area 1 | Other regions | amygdalopiriform transition area |
|  | cingulate cortex, area 2 |  | dorsal intermediate entorhinal cortex |
| Motor | primary motor cortex |  | dorsolateral entorhinal cortex |
|  | secondary motor cortex |  | dorsolateral orbital cortex |
| Retrosplenial | retrosplenial dysgranular cortex |  | ectorhinal cortex |
|  | retrosplenial granular cortex, b region |  | frontal cortex, area 3 |
|  | retrosplenial granular cortex, c region |  | medial entorhinal cortex |
| Somatosensory | primary somatosensory cortex |  | perirhinal cortex |
|  | primary somatosensory cortex, barrel field |  | parietal cortex, posterior area, caudal part |
|  | primary somatosensory cortex, dysgranular zone |  | parietal cortex, posterior area, dorsal part |
|  | primary somatosensory cortex, oral dysgranular zone |  | parietal cortex, posterior area, rostral part |
|  | primary somatosensory cortex, forelimb region |  | ventral intermediate entorhinal cortex |
|  | primary somatosensory cortex, hindlimb region |  |  |
|  | primary somatosensory cortex, jaw region |  |  |
|  | primary somatosensory cortex, shoulder region |  |  |
|  | primary somatosensory cortex, trunk region |  |  |
|  | primary somatosensory cortex, upper lip region |  |  |
|  | secondary somatosensory cortex |  |  |
