# Supplementary material for: Regional Volume Decreases in the Brain of Pax6 Heterozygous Mutant Rats: MRI Deformation-Based Morphometry
Source: PLoS One. 2016 Jun 29;11(6):e0158153. doi: 10.1371/journal.pone.0158153 (PMC4927189; doi:10.1371/journal.pone.0158153)
Supplement: S2 Table — (DOCX) [file pone.0158153.s002.docx]

|  | *rSey^2^/+* rats (female) | | WT rats (female) | | Difference (%) |  |  | *rSey^2^/+* rats (male) | | WT rats (male) | | Difference (%) |  |  |
| --- | --- | --- | --- | --- | --- | --- | --- | --- | --- | --- | --- | --- | --- | --- |
|  | mean (µl) | SD (µl) | mean (µl) | SD (µl) |  | *F* |  | mean (µl) | SD (µl) | mean (µl) | SD (µl) |  | *F* |  |
| Gray matter | 1046.1 | 41.0 | 1091.2 | 51.6 | -4.1 | 5.29 | ***** | 1150.4 | 59.6 | 1181.5 | 50.8 | -2.6 | 2.89 |  |
| White matter | 551.2 | 25.0 | 577.1 | 20.7 | -4.5 | 6.63 | ***** | 579.7 | 34.2 | 595.6 | 23.6 | -2.7 | 2.88 |  |
| CSF | 130.4 | 11.9 | 135.8 | 15.9 | -4.0 | 0.91 |  | 156.3 | 17.4 | 155.1 | 13.1 | 0.7 | 0.05 |  |
| Sum of Gray and white matter and CSF | 1727.8 | 61.2 | 1804.1 | 77.3 | -4.2 | 6.35 | ***** | 1886.3 | 99.9 | 1932.2 | 72.7 | -2.4 | 2.63 |  |
|  |  |  |  |  |  |  |  |  |  |  |  |  |  |  |
| Accumbens nucleus | 11.5 | 0.5 | 11.8 | 0.8 | -2.6 | 1.18 |  | 12.5 | 0.8 | 12.5 | 0.8 | -0.2 | 0.01 |  |
| Amygdala | 37.1 | 1.7 | 39.0 | 1.9 | -4.8 | 8.05 | ****** | 41.0 | 1.8 | 42.7 | 1.6 | -4.0 | 7.46 | ****** |
| Anterior commissure | 2.9 | 0.2 | 3.3 | 0.2 | -12.0 | 34.98 | ******** | 3.1 | 0.2 | 3.3 | 0.2 | -5.9 | 9.61 | ****** |
| Bed nucleus of the stria terminalis | 2.3 | 0.1 | 2.3 | 0.1 | -1.0 | 0.24 |  | 2.5 | 0.1 | 2.5 | 0.1 | 1.0 | 0.31 |  |
| Cerebellum | 266.5 | 11.4 | 270.5 | 12.3 | -1.5 | 0.41 |  | 293.8 | 23.1 | 294.1 | 16.0 | -0.1 | 0.00 |  |
| Cingulum | 4.2 | 0.3 | 4.6 | 0.2 | -8.9 | 18.01 | ******** | 4.7 | 0.3 | 5.0 | 0.2 | -6.1 | 11.15 | ****** |
| Corpus callosum | 64.5 | 4.2 | 70.7 | 3.7 | -8.7 | 17.81 | ******** | 70.0 | 4.5 | 74.0 | 2.8 | -5.3 | 8.42 | ****** |
| Diagonal domain | 4.6 | 0.2 | 4.4 | 0.2 | 3.3 | 3.65 |  | 4.7 | 0.2 | 4.6 | 0.2 | 2.5 | 2.63 |  |
| Diencephalon | 94.3 | 4.1 | 100.2 | 4.2 | -5.9 | 12.18 | ******* | 100.4 | 5.3 | 104.9 | 4.2 | -4.2 | 7.75 | ****** |
| Fimbria | 13.8 | 0.6 | 14.5 | 0.7 | -5.1 | 6.06 | ***** | 14.8 | 1.0 | 15.1 | 0.7 | -2.0 | 1.20 |  |
| Hindbrain | 190.9 | 6.8 | 190.1 | 4.0 | 0.4 | 0.08 |  | 198.4 | 8.7 | 197.2 | 7.8 | 0.6 | 0.21 |  |
| Hippocampal formation | 92.1 | 4.6 | 96.3 | 5.0 | -4.4 | 4.03 | ***** | 104.3 | 7.0 | 107.2 | 5.0 | -2.7 | 2.21 |  |
| Hypothalamus | 32.4 | 1.3 | 33.3 | 1.4 | -2.8 | 2.99 |  | 35.3 | 1.7 | 35.5 | 1.1 | -0.6 | 0.21 |  |
| Internal capsule | 26.8 | 1.4 | 28.2 | 1.2 | -4.9 | 7.88 | ****** | 28.2 | 1.5 | 29.2 | 1.2 | -3.2 | 4.12 | ***** |
| Neocortex | 550.8 | 20.1 | 582.6 | 23.8 | -5.4 | 12.94 | ******* | 603.2 | 25.8 | 627.0 | 22.5 | -3.8 | 8.31 | ****** |
| Midbrain | 83.0 | 3.2 | 87.0 | 5.1 | -4.7 | 4.69 | ***** | 88.9 | 6.3 | 92.5 | 4.4 | -3.9 | 4.16 | ***** |
| Olfactory structures | 110.1 | 3.5 | 115.3 | 4.6 | -4.5 | 12.60 | ******* | 117.9 | 3.5 | 120.4 | 3.6 | -2.1 | 3.37 |  |
| Optic pathways | 3.6 | 0.2 | 3.8 | 0.2 | -5.0 | 9.25 | ****** | 3.8 | 0.2 | 3.9 | 0.2 | -2.6 | 3.24 |  |
| Pallidum | 14.3 | 0.7 | 15.1 | 0.8 | -5.4 | 7.51 | ****** | 15.3 | 0.9 | 15.8 | 0.8 | -2.6 | 2.27 |  |
| Pineal gland | 1.6 | 0.1 | 1.7 | 0.2 | -7.5 | 4.13 | ***** | 1.9 | 0.2 | 2.0 | 0.2 | -8.3 | 7.85 | ****** |
| Pituitary | 10.3 | 0.4 | 10.2 | 0.4 | 1.1 | 0.59 |  | 10.1 | 0.3 | 10.1 | 0.4 | -0.5 | 0.11 |  |
| Preoptic area | 7.5 | 0.3 | 7.6 | 0.4 | -0.7 | 0.12 |  | 8.3 | 0.5 | 8.2 | 0.3 | 1.7 | 0.95 |  |
| Septum | 12.0 | 0.5 | 12.2 | 0.6 | -1.8 | 0.73 |  | 13.0 | 1.0 | 12.9 | 0.5 | 1.1 | 0.34 |  |
| Striatum | 76.2 | 4.3 | 81.2 | 7.6 | -6.2 | 4.32 | ***** | 84.8 | 6.8 | 85.8 | 6.1 | -1.1 | 0.18 |  |
| Substantia | 5.8 | 0.4 | 6.0 | 0.3 | -2.6 | 1.43 |  | 6.1 | 0.4 | 6.3 | 0.2 | -2.7 | 1.99 |  |
| Ventricles | 6.2 | 0.5 | 6.4 | 0.6 | -2.7 | 0.43 |  | 7.5 | 1.1 | 7.3 | 0.4 | 3.1 | 0.83 |  |
| Two-way ANOVA: *p < 0.05, **p < 0.01, ***p < 0.001, ****p < 0.0001  ROI, region-of-interest; *rSey^2^/+*, Pax6 heterozygous mutant; WT, wild-type; SD, standard deviation; CSF, cerebrospinal fluid | | | | | | | | | | | | | | |
