# Supplementary material for: Regional Volume Decreases in the Brain of Pax6 Heterozygous Mutant Rats: MRI Deformation-Based Morphometry
Source: PLoS One. 2016 Jun 29;11(6):e0158153. doi: 10.1371/journal.pone.0158153 (PMC4927189; doi:10.1371/journal.pone.0158153)
Supplement: S3 Table — (DOCX) [file pone.0158153.s003.docx]

|  | *rSey^2^/+* rats (female) | | WT rats (female) | | Difference (%) |  |  | *rSey^2^/+* rats (male) | | WT rats (male) | | Difference (%) |  |  |
| --- | --- | --- | --- | --- | --- | --- | --- | --- | --- | --- | --- | --- | --- | --- |
|  | mean (µl) | SD (µl) | mean (µl) | SD (µl) |  | *F* |  | mean (µl) | SD (µl) | mean (µl) | SD (µl) |  | *F* |  |
| Insular | 39.7 | 1.9 | 41.8 | 2.3 | -5.0 | 7.79 | ****** | 43.4 | 1.8 | 44.7 | 1.9 | -2.9 | 3.32 |  |
| Auditory | 33.8 | 1.9 | 36.3 | 2.1 | -6.7 | 11.27 | ****** | 37.0 | 1.9 | 38.8 | 1.8 | -4.6 | 6.99 | ***** |
| Cingulate | 32.8 | 1.4 | 34.7 | 1.3 | -5.5 | 11.96 | ****** | 35.6 | 1.6 | 37.0 | 1.4 | -3.8 | 7.47 | ****** |
| Motor | 68.2 | 3.1 | 72.1 | 3.2 | -5.4 | 8.95 | ****** | 74.9 | 3.9 | 77.4 | 3.5 | -3.2 | 4.12 | ***** |
| Retrosplenial | 30.6 | 1.0 | 32.4 | 1.2 | -5.4 | 13.35 | ******* | 33.7 | 1.5 | 35.8 | 1.3 | -5.9 | 22.50 | ******** |
| Somatosensory | 123.3 | 5.9 | 131.1 | 6.1 | -5.9 | 12.63 | ******* | 133.6 | 5.4 | 139.2 | 5.7 | -4.0 | 7.52 | ****** |
| Visual | 53.7 | 1.8 | 57.2 | 1.7 | -6.1 | 23.62 | ******** | 58.3 | 2.0 | 61.9 | 2.1 | -5.8 | 28.81 | ******** |
| Association | 17.8 | 0.6 | 19.0 | 0.6 | -6.7 | 23.65 | ******** | 19.4 | 0.8 | 20.4 | 0.7 | -5.1 | 17.93 | ******** |
| Other regions | 84.6 | 2.9 | 87.8 | 3.6 | -3.6 | 5.95 | ***** | 92.5 | 4.0 | 95.3 | 3.1 | -2.9 | 5.15 | ***** |
| Two-way ANOVA: *p < 0.05, **p < 0.01, ***p < 0.001, ****p < 0.0001  ROI, region-of-interest; *rSey^2^/+*, Pax6 heterozygous mutant; WT, wild-type; SD, standard deviation; CSF, cerebrospinal fluid | | | | | | | | | | | | | | |
